# Examine integrating PCM yarns for enhancing merchant maritime uniform fabricated by polyester double cloth fabric

**DOI:** 10.1038/s41598-025-18093-9

**Published:** 2025-09-15

**Authors:** K. M. Seddik, Marwa. A. Ali, Sarah Yahia, Manar Y. Abd El-Aziz

**Affiliations:** 1 Clothing and Knitting Industrial Research Department, Textile Research and Technology Institute, National Research Centre, 33-El-Behouth St., Dokki, P.O. 12622, Giza, Egypt; 2 Spinning and Weaving Engineering Department, Textile Research and Technology Institute, National Research Centre, 33-El Behouth St., Dokki, P.O. 12622, Giza, Egypt

**Keywords:** PCM yarns, Maritime uniforms, Double cloth, Polyester microfibers, Polyester renova, Engineering, Materials science

## Abstract

Functional garments play a crucial role in various professional settings, particularly in merchant maritime uniforms, which must be optimized to meet users’ requirements and withstand environmental fluctuation. In this study, a double-cloth woven structure was developed using phase change material (PCM) yarns, which were strategically incorporated into the backcloth layer intended for direct skin contact. Polyester yarns, including microfibers and Renova variants, were selected for their innovative mechanical and comfort-related properties. Sample designs were guided by the mechanical characteristics of the yarns. Comprehensive evaluations were conducted using standardized test methods to assess physical and mechanical properties, comfort and thermal regulation, UV protection, antistatic behavior, and differential scanning calorimetry (DSC) performance. Statistical analyses—including column bar graphs, one-way ANOVA (*p* ≤ 0.05), and radar charts—demonstrated that the integration of PCM yarns significantly enhanced fabric performance. Key improvements included reduced thickness, increased breathability and water absorbency, and enhanced thermal comfort. Notably, laying viscose PCM yarns outperformed polyester PCM yarns in supporting the produced fabric attitude for the proposed application.

## Introduction

Functional clothing is designed to offer users a specific performance by applying high-performance fibers, unique finishes, or innovative modifications^1^. One of the most important aspects of this clothing is its comfort, which involves multiple techniques^2^. Innovative solutions to enhance the comfort, functionality, and performance of this type of clothing have consistently been sought in various applications^3,4^.

Merchant maritime is one of the occupational types that contacts a wide range of textiles for several applications^5,6^. Among the most demanding textiles in this sector are the uniforms, which should provide not only durability but also resistance to harsh environmental conditions^5,7^. The fabrics used for merchant marine uniforms are made from polyester and acrylic, combined with basic weave structures such as satin, twill, and plain, which supply insufficient protection against fluctuating temperatures and high humidity^8^. Therefore, thermal comfort becomes a crucial element in addressing those challenges and supporting functionality by regulating the heat equilibrium between the human body and the surrounding environment. Embedding new technologies plays a vital role in enhancing several aspects of textile features, whether through weaving processing or utilizing special techniques^7^.

**Fig. 1:**
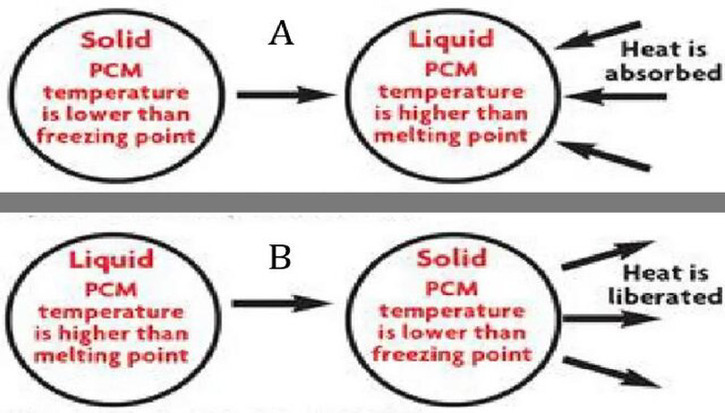
The temperature change PCM^11^.

**Fig. 2:**
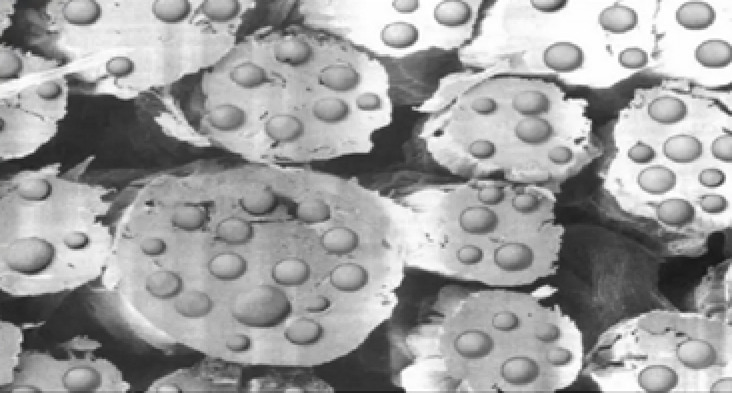
The micro-encapsulated PCM by wet spinning^13^.

Nowadays, the integration of phase change material (PCM) technology into textiles signifies an exciting advancement in many categories of apparel end-uses, such as sportswear, casual wear, safety wear, etc. PCMs are materials that absorb, store, and release thermal energy during phase transitions (from solid to liquid or vice versa). Several researchers have demonstrated that involving PCM yarns in fabric has shown promising results in enhancing the thermal performance of textiles without compromising their strength or comfort properties, thereby providing a more distinguished wearing experience in varying environmental conditions^9,10^. Figure 1 observes the effect of the PCM in adapting temperature^11^. In the same context, the researchers have verified that micro-encapsulated PCM yarns are among the most stable and durable yarns to produce various fabrics, where the paraffinic used are encased in tiny capsules and permanently fixed within the fiber structure during the wet spinning processes of fibers, as displayed in Fig. 2^12,13^. Additionally, using such innovative technology aligns with growing trends in sustainability, as many PCMs are derived from bio-based sources, further enhancing the eco-friendliness of such textile solutions^11,14^.

These notable advancements indicate that blending PCM technology with other materials could redefine the standard of comfort and functionality in the context of merchant maritime uniforms, where the workers are often exposed to extreme temperature fluctuations indoors and outdoors.

Polyester material has long been a staple in the production of uniforms due to its excellent qualities, which include dimensional stability, excellent resistance to most organic solvents, and perfect high tenacity as well^15,16^. Moreover, easy care, quick drying, and strong heat resistance^17,18^. Renova is a type of polyester yarn that closely resembles wool in appearance and texture. Moreover, Renova yarns are characterized by their softness, well-sized, dry texture, stretchiness, and warm feeling^19,20^. Microfiber is another type of polyester yarn, which has recently been used in various apparel applications, thanks to its unique qualities. References suggest that this polyester yarn type can serve as a viable alternative to cotton^21^. Furthermore, it strongly impacts the surface and mechanical characteristics of fabrics, associating the movement of filaments within a cross-section with elasticity, comfort, and dimensional stability^22,23^. Also, microfibers are used in many different aspects owing to their unique qualities and enormous potential for both practical and aesthetic trends^24,25^.

Previous studies found that different weave structures affected fabrics’ comfort and thermal properties^26^. In terms of achieving two different weave layers of fabrics, the double cloth weave structure can play a crucial role. This weave structure is characterized by the incorporation of two independent sets of warp and weft, which collectively form the face and back layers of the fabric. Each layer can be identified by a specific woven structure, which imparts distinct properties such as thermal insulation^27^. The differentiation between the facecloth and backcloth has the potential to enhance the fabric’s performance according to the intended application^28^. Thus, it is suitable for use in garments exposed to various environmental conditions.

According to all the above, the research aims to leverage the double cloth weave structure characterizations by incorporating different phase change material yarns to enhance merchant maritime uniforms. Polyester microfiber is used as a warp yarn, while polyester Renova and phase change materials (PCMs) are used as weft yarns, which are the main keys of the fabricated samples. The innovation approach correlated to the weft materials insertion in the inner layer (backcloth) that is supposed to be in contact with the body skin, where two types of phase change materials yarns (PCM polyester and PCM viscose) were utilized, as well as the conventional polyester (Renova). In the meantime, the facecloth that is supposed to be exposed to different environmental conditions is limited to polyester Renova for all the produced samples.

## Experimental work

**Fig. 3:**
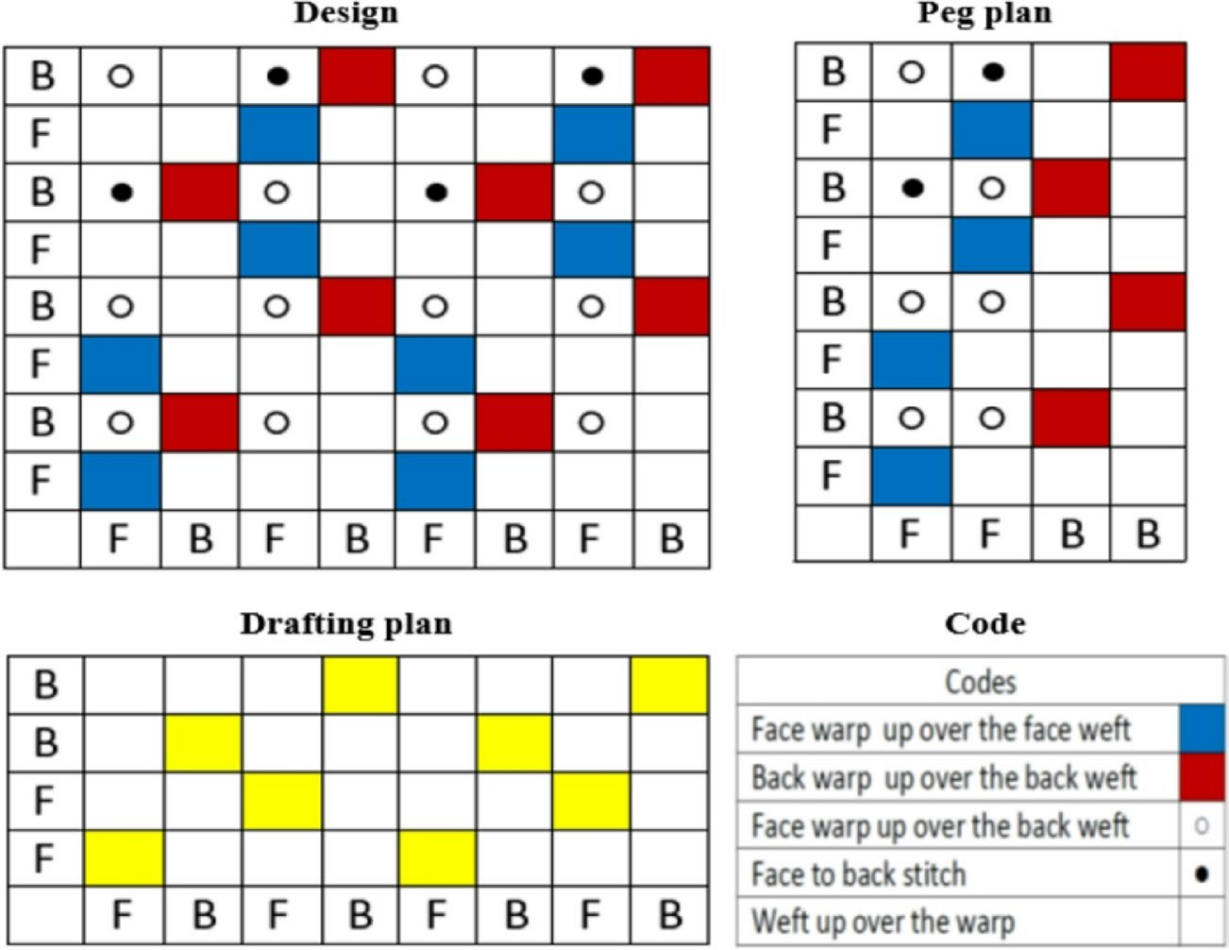
Double-cloth weave structure of the produced samples.

Three samples were fabricated incorporating two thermally active materials, based on Outlast technologies (paraffinic phase change material within polyester and viscose yarn structures). All samples were executed with the double-cloth weave structure (two layers), where a warp rib 2/2 structure for the facecloth and a plain 1/1 structure for the backcloth, as explicated in Fig. 3.

The warp material for all samples was polyester microfibers with a yarn count of 150/1 denier (consisting of 288 filaments at cross-section). At the same time, the weft materials were polyester Renova with a yarn count of 160/1 denier (1500 twists/meter) and PCMs (polyester and viscose) with a yarn count of 170/1 denier. The variability of the weft insertion occurred on the backcloth only (which is supposed to be in contact with the body skin). While the facecloth was exclusively Renova for all samples. Table 1 exhibits the scheme of the produced samples.

**Table 1:** Scheme of fabricated samples specifications.

Sample code	Warp yarns	Weft yarns	
		(Face cloth)	(Back cloth)
1	Polyester microfiber	Renova	100% Renova
2			100% Polyester PCM
3			100% Viscose PCM

All samples were manufactured utilizing the same weaving loom machine specifications as presented in Table 2 and flattened on the cutting table in a relaxed state to release any tension.

**Table 2:** Weaving loom machine specifications.

Item	Describe
Machine mark/model	Picanol/2008
Weft insertion	Rapier
Shedding mechanism	Electronic Dobby
Machine speed (rpm)	600
Machine Width (cm)	170
Warp density Ends/cm	60
Weft density Picks/cm	40
Reed (dents/cm)	15
Sley (No. of yarn per dent)	4
No. of heald frames	8 (fabric) + 2 (selvages)
Accumulators	4
warp beam	1
cloth beam	1
Color selector	8 color

### Laboratory tests

Under standard test methods, multiple tests were operated for used yarns and produced samples as follows:

A) **Yarn evaluation**:

To recognize the mechanical characteristics (force resistance) of employed yarns, several tests were undertaken. For the PCMs yarns that serve a crucial role in providing comfortability at various aspects of the produced fabrics, the test entails; time to break [s], breaking force [gF], tenacity [g/den], elongation [%], and breaking work [gF.cm] was occurred according to (ASTM D1578)^29^. While for polyester microfiber and polyester Renova yarns, the test was conducted to assess the mechanical properties at the break, like load [kgf], tenacity [g/den], elongation [%], and displacement [mm] according to (ASTM D2256)^30^.

B) **Fabric samples evaluation**.

Various tests were carried out to genuinely understand the impact of different variables on sample characterizations. The testing is classified into three categories:

- Physical and mechanical characteristics include areal density [g/m^2^] according to (ASTM D3776)^31^, thickness [mm] according to (ASTM D1777)^32^, tensile strength [Kgf/mm^2^], and elongation [%] according to (ASTM D5035)^33^.
- Comfort and thermal characteristics include air permeability [cm^3^/cm^2^.s] according to (ASTM-D737-18)^34^, water permeability [l/cm^2^.s] according to (ASTM D4491/D4491M-22)^35^, thermal conductivity [w/cm.c^o^] according to (ASTM C518)^36^, and Differential Scanning Calorimetry (DSC) according to (ASTM E793)^37^ by using DSC131 evo [SETARAM Inc., France]. The instrument was calibrated using the standards (Mercury, Indium, Tin, Lead, Zinc, and Aluminium). Nitrogen and Helium were used as the purging gases. The test was programmed, including the heating zone from − 15 °C to 50 °C with a heating rate of 5 °C/min. The samples were weighed in an aluminium crucible (120 µL) and introduced to the DSC^38^. The thermal storage per square meter was collected through Eq. 1^[39]^ as follows.

1 $${\mathbf{Thermal}}{\text{ }}{\mathbf{storage}}/{{\mathbf{m}}^{\mathbf{2}}}\,=\,{\mathbf{Heat}}{\text{ }}{\mathbf{Latent}}{\text{ }} \times {\text{ }}{\mathbf{Areal}}{\text{ }}{\mathbf{Density}}/{{\mathbf{m}}^{\mathbf{2}}}$$

Protective characteristics include ultraviolet protection factor (UPF) according to (AATCC-183)^40^ and electrostatic charge [Kv] according to (ISO 18080-1)^41^.

Before testing, all samples were placed in a relaxed state and conditioned for 24 h in a standard atmosphere according to (ISO 139)^42^; the test method was a temperature of 20 ± 2 °C and relative humidity of 65 ± 4%.

### Data analyzing

Three replicates were measured for each sample at different tests; all readings were collected and tabulated, and the mean values were calculated. The findings were plotted using a column chart. Furthermore, the ANOVA test at *p-value* ≤ 0.05 was analyzed to define the significant/non-significant effect of the variables on manufactured sample characteristics. Eventually, the radar chart areas were computed and evaluated to demonstrate the best sample performance by calculating the quality factor using the following Eq. 2^43^.

2 $$\:\text{Q}\text{F}\:=\frac{\mathbf{X}}{{\mathbf{X}}_{\mathbf{m}\mathbf{a}\mathbf{x}}}\:\times\:100\:\text{o}\text{r}\:\text{Q}\text{F}\:=\frac{{\varvec{X}}_{\varvec{m}\varvec{i}\varvec{n}}}{\varvec{X}}\:\times\:100$$

**Where** X (sample reading separately), X_Max_ (highest reading), and X_Min_ (lowest reading).

## Results and discussion

### Yarn characteristics results

A) **PCMs Yarns**

Based on the mechanical properties statistics as shown in Table 3. The results indicate that despite each of the utilized PCM yarns having almost identical mechanical qualities, a relative difference can be found. Wherein, polyester PCM yarns realize an advantage over viscose PCM, especially on elongation [%] and tenacity [g/den] (which is normally applied to determine stretch ability and load resistance of individual yarns) that in turn influences the time to break [sec] and break force [gF]. This difference can be attributed to the nature of the chain linking for the polymer shells that enclose the PCM materials, as it can be developed in the synthetic (polyester) base more than the natural (viscose) base, affecting yarn characteristics that reflect on the mechanical resistance performance. In the same vein, the results imply that break work values, which are crucial for various fabric-manufacturing processes, are increased with using polyester PCM compared to viscose PCM.

**Table 3:** Mechanical property of the utilized PCMs yarns.

Stats	Viscose PCM (30% Viscose Outlast, 70% Down)					Polyester PCM (30% Polyester Outlast, 70% Down)				
	Time to break [sec]	B-Force [gF]	Elong. [%]	Tenacity [g/den]	B-Work [gF.cm]	Time to break [sec]	B-Force [gF]	Elong. [%]	Tenacity [g/den]	B-Work [gF.cm]
Mean	2.00	624.6	13.31	3.59	2302	2.07	639.8	13.79	3.67	2226
Min.	1.73	575.0	11.47	3.29	1773	1.81	554.9	12.05	3.19	1584
Max.	2.22	662.7	14.76	3.80	2678	2.24	700.7	14.90	4.02	2752

B) **Polyester Yarns**

Table 4 outlines the mechanical properties of the employed polyester yarns. The results demonstrate that polyester microfiber yarn is more featured in elongation than polyester Renova. This could be returned to the displacements of yarns that reached 64.340 mm and 53.301 mm, respectively, which had a positive effect on the yarns’ extension and thus on the elongation percentage. Furthermore, the results assign that polyester Renova yarn offers more tenacity than polyester microfiber yarn, as evidenced by the load at a breaking point where the recorded averages (mean) are 0.669 [Kgf] and 0.639 [Kgf], respectively, indicating that polyester Renova yarns possess enhanced resistance to force compared to their microfiber counterparts^44^. The justification can be attributed to the filament composition, as the twisting generated among the fibers that reached 1500 twists /m aids in minimizing movement and builds up an empowered strength due to heightened friction.

**Table 4:** Mechanical property of the utilized polyester yarns.

Stats	Microfibers				Renova			
	Load [Kgf]	Tenacity [g/den]	Displacement [mm]	Elong. [%]	Load [Kgf]	Tenacity [g/den]	Displacement [mm]	Elong. [%]
Mean	0.639	4.459	64.340	32.170	0.669	9.123	53.301	26.650
Min.	0.614	4.245	53.518	26.759	0.637	8.768	45.493	22.747
Max.	0.664	4.625	70.983	33.492	0.694	9.484	58.582	29.291

Based on the above characteristics, the schemes of fabricated samples were designed as shown in Table 1, considering the combination of elasticity and durability in promoting the target of the final end-use application.

### Samples characteristics results

A) **Physical and Mechanical Characteristics**

**Fig. 4:**
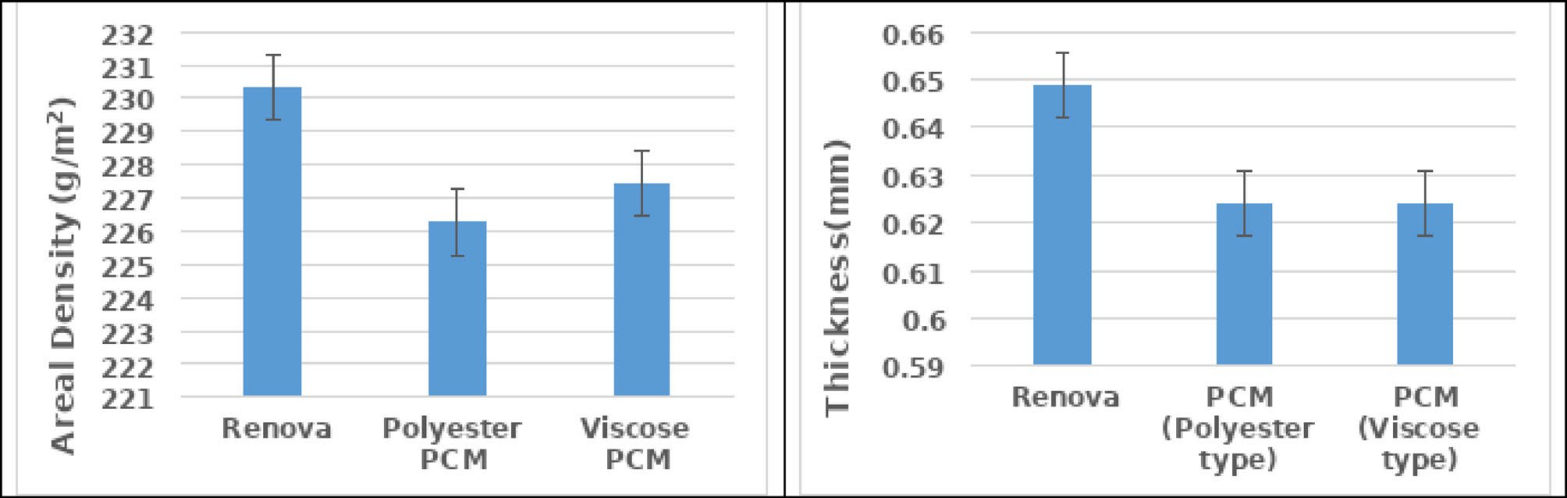
The physical characteristics of the produced samples.

The findings of the areal density (weight) and thickness properties pointed out that even though polyester Renova yarns and PCM yarns have approximately the same count number, there are sizeable variations in the physical characteristics of the manufactured samples. Utilizing PCM yarns tends to decrease the weight per unit area and the height of the samples in comparison to polyester Renova yarns. The explanation could be traced to the impact of the twist processing (1500 twists/meter) on the yarn packing density and diameter properties, which facilitates increasing the weight and the thickness of the produced samples. Moreover, the findings indicate that integrating polyester PCM contributes to providing a lighter weight and lower thickness for the produced samples than using viscose PCM. Figure 4 illustrates the properties of the areal density (weight) and thickness of the produced samples.

**Fig. 5:**
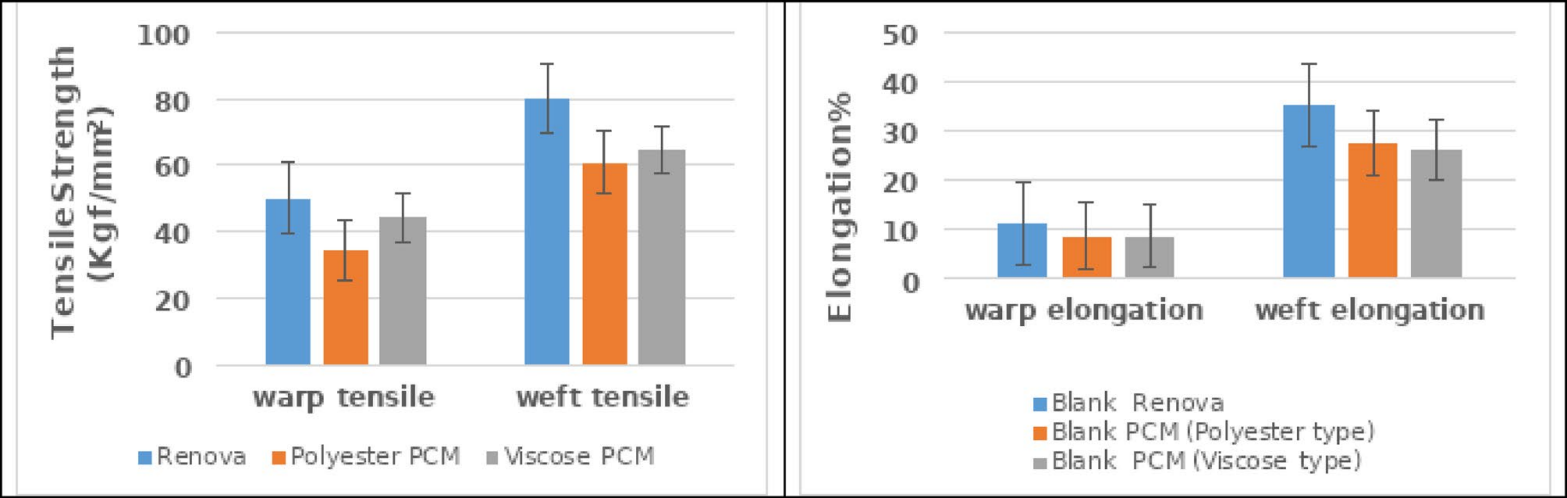
The mechanical characteristics of the produced samples.

Additionally, the findings indicate that involving polyester Renova yarns aggregates the mechanical properties of the produced samples, as shown in Fig. 5. Where the data reveal an increase in the tensile strength [kgf/mm^2^] and elongation [%] by using polyester Renova when compared to the phase change material (PCM) yarns. The interpretation could be correlated to the mechanical properties of each yarn as presented in Tables 1 and 2, in which polyester Renova compromises the highest tenacity and elongation compared to PCM yarns.

In the same context, the results indicate that the incorporation of PCM viscose yarns provides a greater enhancement in the strength resistance of the produced samples, even though the high tenacity [g/den] was linked to PCM polyester yarns. The explanation may be closely related to the intrinsic properties of PCM yarn composition. As viscose with cellulose-based, which is naturally hydrophilic, tends to saturate with moisture, leading to create more shrinkage in the produced sample, particularly during relaxation. This results in enriched interlacing among yarns per unit area, and thus increases interfacial friction, which plays a crucial role in improving the overall resistance to breaking load.

On the other hand, the findings shed light on the fact that the elongation property (stretch-ability) is primarily aligned with the inherent extensibility attribute of the PCM yarns, which is elevated with the use of polyester PCM instead of viscose PCM, as elucidated in Table 3.

B) **Comfort and Thermal Characteristics**

**Fig. 6:**
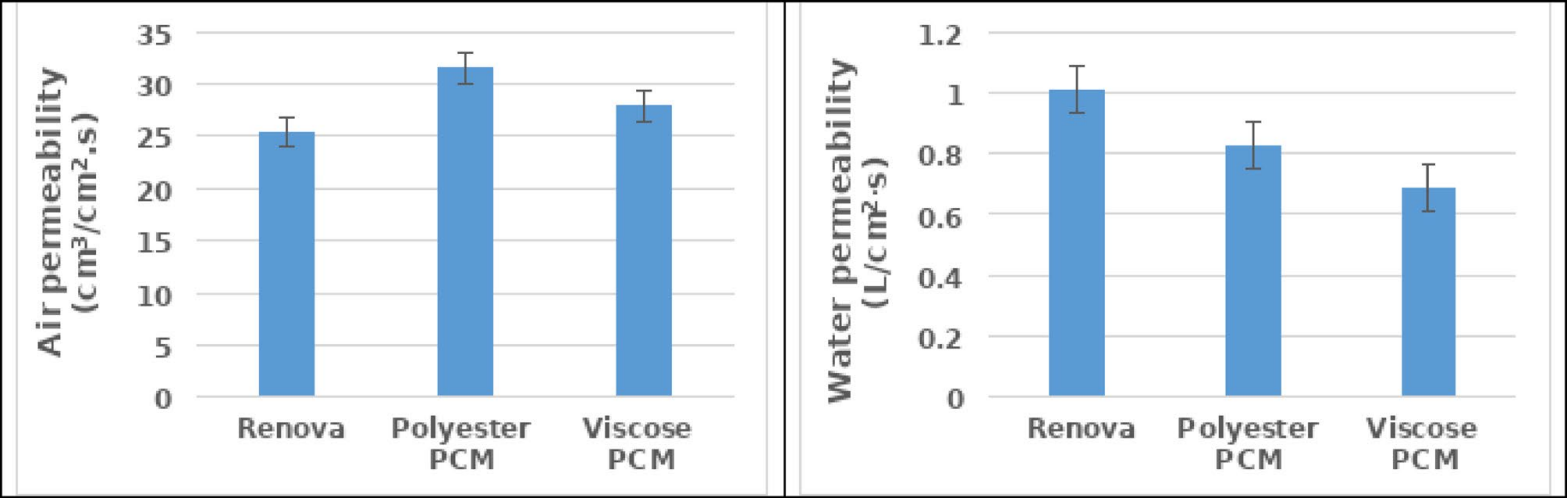
The comfort characteristics of the produced samples.

Figure 6 demonstrates the comfort characteristics of the produced samples. The findings denote that utilizing phase change material yarns assists in improving comfort properties of produced samples from them, as their breathability and absorbency are increased compared to the sample fabricated from conventional polyester yarns. In addition, the findings clarify that integrating polyester PCM yarn improves the airflow ability within a sample, while viscose PCM develops the water absorbency. The justification could be traced to the compactness phenomenon, which was related powerfully to viscose PCM yarns due to its base nature, resulting in a porosity reduction of the woven sample and hence decreasing the permeability of air and water (i.e., less breathable and high absorption)^[45]^.

**Fig. 7:**
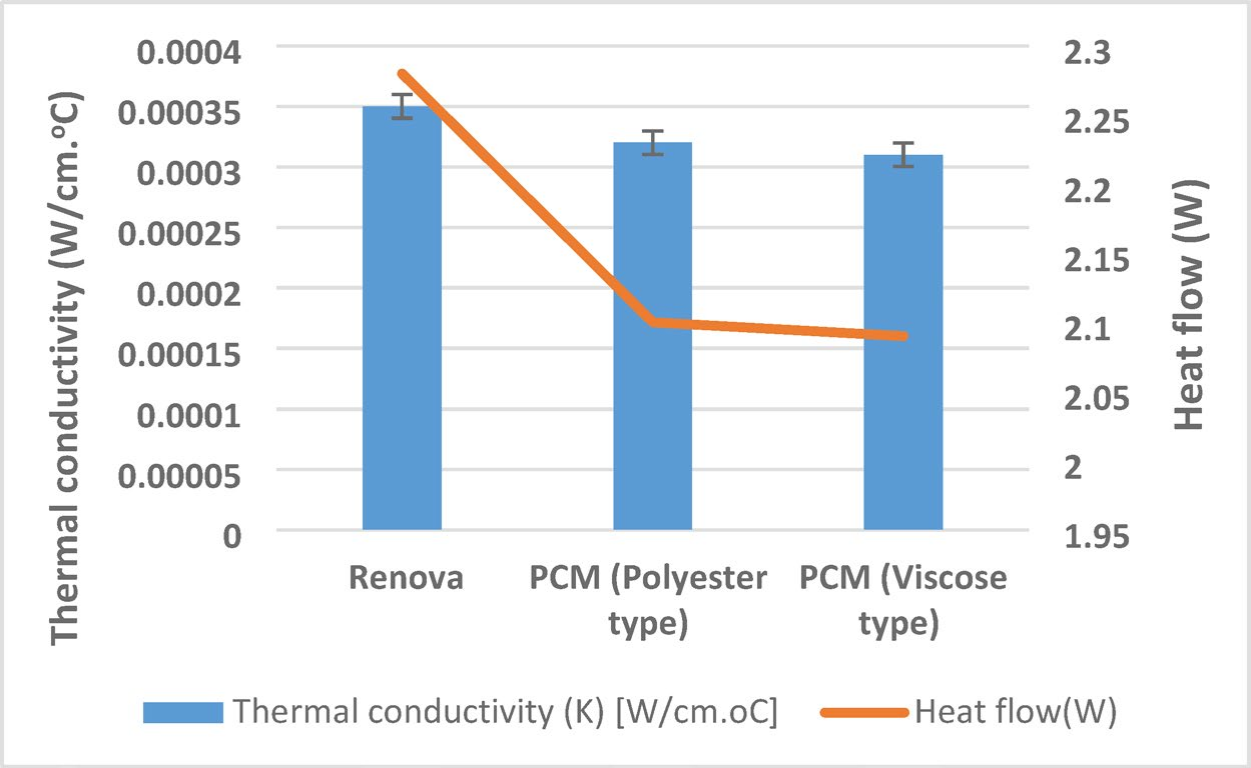
The thermal characteristics of the produced samples.

Regarding thermal comfort, each of the thermal conductivity and heat flow was conducted as shown in Fig. 7. The findings clarify that the sample engaged with polyester Renova accomplished lower thermal resistivity and higher heat flux, unlike other samples that integrate with PCM yarns, highlighting the remarkable capability of its thermal control. Besides, the findings declare the viscose PCM yarns obtained a relative reduction in thermal conductivity and heat flow compared to polyester PCM, which could be traced to the increasing of thermally active material per unit area on the sample of viscose PCM yarns due to shrinkage (in a relaxed state), as proved in the areal density property in Fig. 4.

C) **Protection Characteristics**

**Fig. 8:**
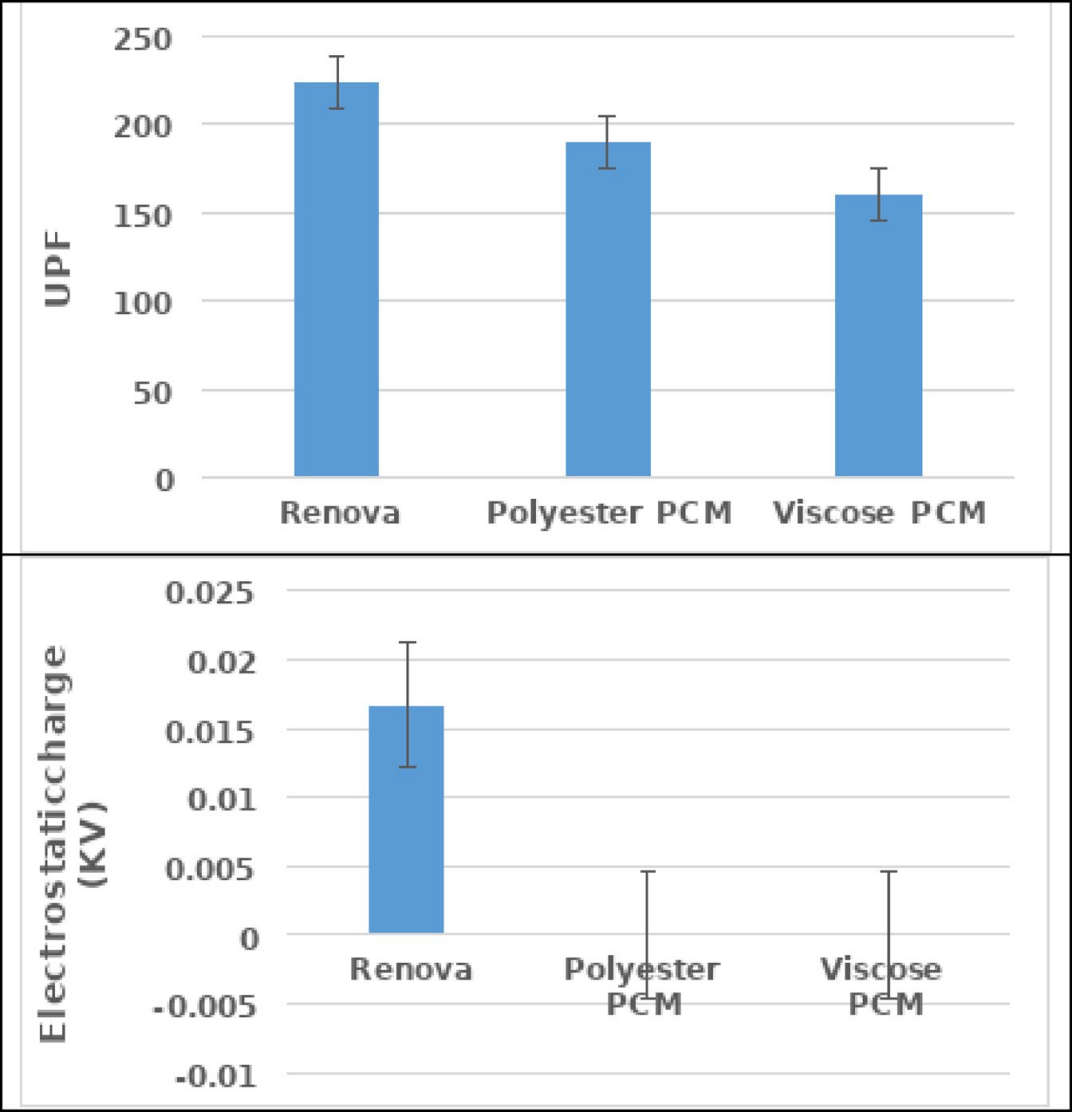
The protection characteristics of the produced samples.

Figure 8 illustrates the protection characteristics of the produced samples in terms of ultraviolet protection factor (UPF) and electrostatic charge. The findings reveal that inserting polyester Renova provides more potential for blocking UV radiation than PCM yarns. The explanation could be linked to increasing yarn density/unit area, which is reflected in weight and thickness values as shown in Fig. 4, which aids the reflection of UV rays, leading to a raised UPF value and increased sun protection. Conversely, the findings mention that PCM yarn insertion magnifies the elimination of electrostatic charge risks (even if it is used in the backcloth only of the double weave fabric) rather than conventional polyester yarn. This may be attributed to their capability to retain water within the produced sample, which helps dissipate electrostatic charges, as shown in Fig. 6.

### Influence of variables on the characteristics of produced samples

The ANOVA test was conducted with a significance level set at a *p-value* ≤ 0.05 to examine the effectiveness of the weft variables on the different examined characteristics, as detailed in Table 5. The findings state that integrating PCM yarns caused a substantial effect on several attributes of the produced samples. Based on the reported *p-values* of each property tested, it can be recognized that the surfaces height of the samples (thickness), the resistance to the break load (tensile strength), thermal conductivity, as well as the UPF and electrostatic chargewere all significantly affected, presenting a pronounced change in sample behavior after consolidating PCM yarn insertion. Additionally, comparing the significantly affected properties, it can be found that the constituting material of the shells on the PCM yarns had a more generous influence than the encapsulated thermal active material, especially on mechanical resistance (weft direction) and thickness results.

**Table 5:** The significant effect of variables on different properties.

Characteristics	*P*-value	Significant effect / Non-Significant effect
Areal Density (g/m^2^)	0.170968	Non-Significant
Thickness (mm)	0.003836	Significant
Warp Tensile (kgf/mm^2^)	0.010817	Significant
Weft Tensile (kgf/mm^2^)	0.002665	Significant
Warp Elongation (%)	0.058121	Non-Significant
Weft Elongation (%)	0.058121	Non-Significant
Air Permeability (cm^3^/cm^2^.s)	0.547169	Non-Significant
Water Permeability (L/cm^2^.s)	0.601148	Non-Significant
Thermal Conductivity [W/cm.^o^C]	0.006592	Significant
UPF	0.013567	Significant
Electrostatic Charge (KV)	0.034114	Significant

### Samples performance

**Fig. 9:**
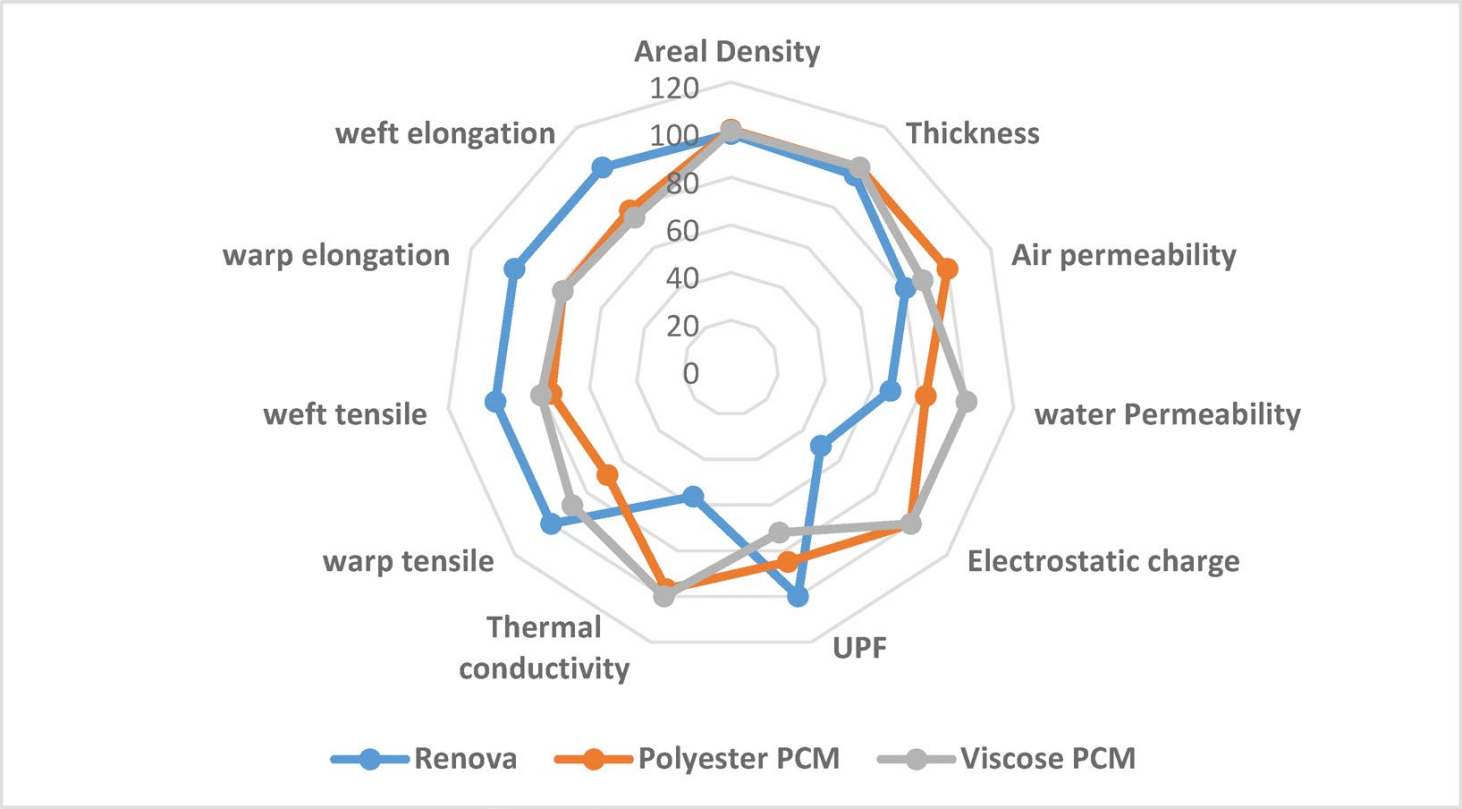
The radar chart area of the produced samples.

The radar chart area was plotted and calculated to identify the preferred performance among produced samples, as offered in Fig. 9; Table 6. The findings confirm that inserting PCM yarn improves the overall performance of the manufactured samples, attending to the final end-use application (maritime uniforms), where the samples produced with PCM yarn integration obtain a higher radar area compared to the sample with Renova yarn. Meanwhile, the findings demonstrate that using viscose PCM yarn can enrich the double-cloth woven fabric attitude to polyester PCM.

**Table 6:** Quality factor for each tested property of the produced samples.

Characteristics	Renova	Polyester PCM	Viscose PCM
Areal Density	98.24	100.00	99.49
Thickness	96.15	100.00	100.00
Air Permeability	80.74	100.00	88.57
Water Permeability	67.73	82.71	100.00
Electrostatic Charge	50.00	100.00	100.00
UPF	100.00	84.93	72.10
Thermal Conductivity	56.36	96.88	100.00
Warp Tensile	100.00	68.71	88.21
Weft Tensile	100.00	76.20	80.65
Warp Elongation	100.00	77.70	77.70
Weft Elongation	100.00	78.57	75.00
Radar area	**22209.90**	**22986.87**	**23672.70**

### Differential scanning calorimetry (DSC)

The latent heat of the produced samples was measured as shown in Table 7. The findings clarify that incorporating PCM yarns improves the thermal regulating capability of the woven samples, where the latent heat content achieves 0.354 [J/g] and 0.420 [J/g] on the samples with polyester PCM and viscose PCM, respectively, while it is not applicable through using Renova. This assigns the impact of encapsulated thermal active material on controlling transition enthalpy, consequently triggering the human body’s sense of comfort. Moreover, the findings indicate that inserting viscose PCM provides a higher propensity to heat capacity than polyester PCM, which could be attributed to the difference between the base nature of the utilized PCM yarns that play an important role in their thermo-physical properties.

In the same context, to anticipate the heat performance of the produced samples, the thermal storage per square meter was collected through Eq. 1^[39]^. The scores affirm that the sample with viscose PCM has a greater ability to enrich comfort owing to thermal sensation than the sample with polyester PCM.

**Table 7:** Differential scanning calorimetry of the produced samples.

Samples	Latent Heat [J/g]	Heat storage of woven samples [J/m^2^]
Renova	Not Applicable	Not Applicable
Polyester PCM	0.354	80.098
Viscose PCM	0.420	95.517

## Conclusion

Based on different laboratory testing and statistical analysis, the findings designate that embedding PCM yarns within fabricated double-cloth polyester fabrics contributes to enhancing several properties that serve merchant maritime uniforms to encounter a variety of occupational challenges that require adaptability with different atmospheres to preserve human body temperature simultaneously maintaining durability and comfort. The following points summarize the results obtained:

- Incorporating PCM yarns on the double-cloth woven samples (even if in the backcloth only) tends to decrease the weight per unit area and thickness height. Furthermore, PCM yarns progress the abilities of airflow, water absorbency, thermal comfort, and electrostatic discharging.
- By comparing PCM yarn types, Inserting the polyester type gave more ability to decrease the areal density and thickness of the produced fabric sample, as well as improve the air permeability and UV blocking, while using the viscose type achieved more capability of supporting tensile strength properties, water absorption, and reducing the thermal conductivity of the produced fabric sample.
- According to the ANOVA test, there are significant differences between the produced samples on several characteristics. Moreover, regarding the radar chart areas, PCM yarns enhance the entire performance of manufactured samples, particularly using viscose type.
- Relating to the DSC results, integrating PCM yarns develops thermal regulation, considering that the viscose type has a great implementation when compared to the polyester type.

## Data Availability

No datasets were generated or analysed during the current study.
